# Cardiovascular-kidney-metabolic syndrome: A new frontier or simple rebranding?

**DOI:** 10.1371/journal.pmed.1004723

**Published:** 2025-08-21

**Authors:** Jennifer Manne-Goehler, Willem Daniel Francois Venter, Mohammed K. Ali, Juan V. A. Franco

**Affiliations:** 1 Division of Infectious Diseases, Brigham and Women’s Hospital, Harvard Medical School, Boston, Massachusetts, United States of America; 2 Medical Practice Evaluation Center, Massachusetts General Hospital, Boston, Massachusetts, United States of America; 3 Ezintsha, Faculty of Health Sciences, University of the Witwatersrand, Johannesburg, Gauteng, South Africa; 4 Emory Global Diabetes Research Center, Robert W. Woodruff Health Sciences Center, Emory University, Atlanta, Georgia, United States of America; 5 Department of Family and Preventive Medicine, Emory University School of Medicine, Atlanta, GeorgiaUnited States of America; 6 Institute of General Practice, Heinrich Heine University Düsseldorf, Düsseldorf, Germany

## Abstract

Cardiovascular-kidney-metabolic (CKM) syndrome is an emerging framework proposed by the American Heart Association for management of patients with cardio-metabolic multimorbidity. This novel framework offers several improvements over existing paradigms; however, it remains unclear whether it represents a new frontier, or a simple rebranding of known clinical principles.

Cardiovascular-kidney-metabolic (CKM) syndrome is an emerging framework proposed by the American Heart Association (AHA) for management of patients with cardio-metabolic multimorbidity that spans major organ systems (Fig 1) [1]. This syndrome aims to capture interactions among metabolic risk factors, chronic kidney disease (CKD), and the cardiovascular system that ultimately lead to poor health outcomes [1,2]. Importantly, this paradigm incorporates a wide range of specific pathologies, including both obesity and diabetes, CKD, and either the risk or presence of heterogeneous forms of cardiovascular disease (CVD), including heart failure, atrial fibrillation, coronary heart disease, stroke, and peripheral artery disease. The pathophysiology of CKM syndrome is rooted in excess or dysfunctional adiposity and upstream behavioral risk factors (sedentary lifestyles, unhealthy diets), and is strongly influenced by social determinants of health (SDOH), while giving a relatively singular nod to the critical role of the kidneys in this pathophysiologic milieu [1,2]. The creation of the CKM health framework was borne of a growing recognition of the interconnectedness of these major organ systems and with a goal to increase early detection and risk reduction.

**Fig 1:**
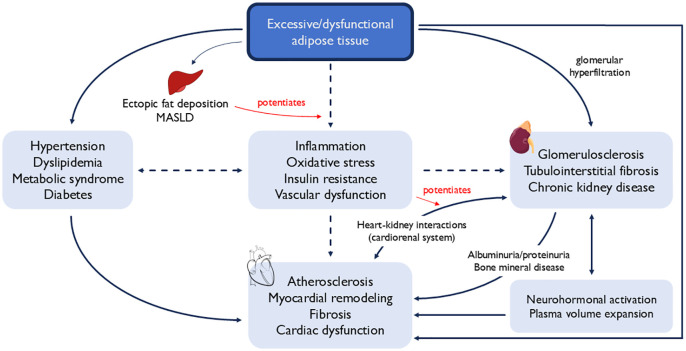
The pathophysiology of cardiovascular-kidney-metabolic (CKM) syndrome. Schematic showing the CKM health framework as proposed by the AHA [2]. Dysfunctional adipose tissue is an important underlying factor in the pathophysiology of CKM syndrome, eventually leading to derangement in these organ systems.

CKM syndrome offers several improvements over existing paradigms for understanding the connections between these major organ systems, with potential future implications for epidemiological estimation, as well as patient-level risk assessment and clinical management. By incorporating unhealthy adiposity within these related conditions and including SDOH in both the etiology and management of these interconnected health states, CKM syndrome offers a modest improvement in the way we understand human health. Indeed, it helps articulate an individual’s total risk as some adiposity can remain preclinical, while other adiposity can have co-existing morbidities [3]. Other potential benefits of the use of CKM syndrome include that, per the AHA, it provides “a framework for optimizing CVD risk reduction and selecting cardioprotective antihyperglycemic agents, such as the sodium-glucose transport protein 2 inhibitors (SGLT2i) or glucagon-like peptide-1 (GLP-1) receptor agonists.” [1,2]. These medicines also offer kidney protection in patients with CKD due to diabetes (GLP-1) and even among those without diabetes (SGLT2i). The CKM framework makes the kidney more central to this pathophysiology and clinical management paradigms. This includes going beyond estimated glomerular filtration rate (an indicator of kidney function) in those with CKD, diabetes, hypertension, and metabolic syndrome to include measurement of urine albumin–creatinine ratio. This helps to more holistically assess CKD and CVD risk, particularly in heart failure. Finally, CKM syndrome may offer improvements in the implementation of care for CKM multimorbidity, including easing care coordination and reimbursement or allowing single digital decision tools to facilitate multiple clinical risk reduction decisions.

However, CKM syndrome has several key impediments to real-world use, leaving questions about what it will ultimately add to current paradigms and approaches. First, the best way to implement this concept in clinical settings remains an unanswered challenge, much like the ‘metabolic syndrome’ before it. This includes a lack of truly feasible risk prediction equations for key health outcomes that are implementable across different settings; though the AHA recently created the Predicting Risk of Cardiovascular Disease Events (PREVENT) equations, which offer a first step in this direction [4]. Another issue is the limited understanding of how CKM syndrome should be treated in the context of a rapidly growing arsenal of possible medical therapies, including SGLT2is, statins, GLP-1 receptor agonists, and more. To address this, the AHA has forthcoming guidelines [5]. Second, while the attention to SDOH is welcome, there is a dearth of concrete guidance on how to measure these SDOH elements among the possible ‘shopping list’ of questions, the validity of SDOH measurement across countries, which interventions to pursue to address these conditions, and how they link to treatment algorithms with common medical therapies [6,7]. Third, from a practical epidemiological perspective, it is unclear whether all facets of CKM would be measurable in many contexts, especially outside but even within high-income settings [8]. In addition, strongly related components of multimorbidity such as mental health are not explicitly captured, such that it may be seen to be inferior to existing frameworks in that distinct but related field. Finally, the CKM syndrome definition presently pays minimal attention to the liver, including metabolic dysfunction-associated steatotic liver disease, despite a growing recognition of this condition’s clear overlap with the other CKM components—prompting the question by some, “should this instead be the CKLM (i.e., adding liver) syndrome?” [9].

In a recent PLOS Medicine study [10], Tsai and colleagues make a worthy effort to operationalize this concept in an epidemiological analysis, charting its prevalence and linkage to mortality risk in a large retrospective cohort of 515,602 participants in Taiwan. The participants were ≥20 years old participants in a health screening program conducted between 1996 and 2017. They assessed the associations between CKM components and the outcomes of all-cause mortality, CVD mortality, and cause-specific mortality over a median follow-up period of 16.5 years, finding that each additional CKM component was associated with a 22% increase in the risk of all-cause mortality and a 37% increase in the risk of CVD mortality compared with those without any CKM components. Moreover, each additional component reduced average life expectancy by 3 years. These epidemiological findings regarding the health impact of considering these conditions together is clearly significant.

Hence, while it may be easy to criticize this proposed approach to tying up a complex myriad of clearly overlapping disease processes, the goal to promote a coherent medical framework by which health professionals may screen for and treat more than one condition or organ system at a time is also laudable, especially given the potential impact of these component conditions on the most critical of health outcomes [11]. Witness the gross under-identification of something as simple as raised blood pressure, and inability to treat to targets, even in the best of health systems, and one can appreciate the significant improvement in care that would result if ‘CKM syndrome’ could be mastered as an integrated concept in clinical contexts.

However, whether CKM syndrome ultimately comes to be viewed as a new frontier, or a simple rebranding or medicalizing of risk with little attention to prevention and changing health behaviors, will depend on how much investment is made in operationalizing this concept. If it leads to more effective awareness and prevention efforts, better risk prediction and tailored but implementable clinical guidelines, it may spark progress. If not, CKM may simply be remembered as a repackaging of what we have long known: that chronic CKLM diseases often co-occur and reinforce one another on the path to poor clinical outcomes.

## Abbreviations

**:**
AHA: American Heart Association
CKD: chronic kidney disease
CKM: cardiovascular-kidney-metabolic
GLP-1: glucagon-like peptide-1
PREVENT: Predicting Risk of Cardiovascular Disease Events
SDOH: social determinants of health
SGLT2i: sodium-glucose transport protein 2 inhibitors
